# Analyzing the resting state functional connectivity in the human language system using near infrared spectroscopy

**DOI:** 10.3389/fnhum.2013.00921

**Published:** 2014-01-29

**Authors:** Behnam Molavi, Lillian May, Judit Gervain, Manuel Carreiras, Janet F. Werker, Guy A. Dumont

**Affiliations:** ^1^Department of Electrical and Computer Engineering, University of British ColumbiaVancouver BC, Canada; ^2^Department of Psychology, University of British ColumbiaVancouver, BC, Canada; ^3^Laboratoire Psychologie de la Perception, Université Paris Descartes, Sorbonne Paris CitéParis, France; ^4^Laboratoire Psychologie de la Perception, Centre national de la recherche scientifiqueParis, France; ^5^Basque Center on Cognition Brain and LanguageSan Sebastián, Spain; ^6^IKERBASQUE, Basque Foundation for ScienceBilbao, Spain

**Keywords:** functional near-infrared spectroscopy, resting state functional connectivity, phase synchronization, language network, BOLD signal

## Abstract

We have evaluated the use of phase synchronization to identify resting state functional connectivity (RSFC) in the language system in infants using functional near infrared spectroscopy (fNIRS). We used joint probability distribution of phase between fNIRS channels with a seed channel in the language area to estimate phase relations and to identify the language system network. Our results indicate the feasibility of this method in identifying the language system. The connectivity maps are consistent with anatomical cortical connections and are also comparable to those obtained from functional magnetic resonance imaging (fMRI) functional connectivity studies. The results also indicate left hemisphere lateralization of the language network.

## 1. Introduction

Functional Near Infrared Spectroscopy (fNIRS) is an optical method for brain functional imaging. Using near infrared light with high penetration depth in human tissue, fNIRS can measure the neuronal activity in response to a task or an environmental stimulation through neurovascular coupling in superficial areas of the brain. fNIRS has been used for functional studies as a portable, less expensive and less restraining alternative to functional Magnetic Resonance Imaging (fMRI) in different task based brain functional studies. One of the more recent areas of interest in both fNIRS and fMRI is the study of interaction between different cortical areas through their intrinsic neuronal signaling (Biswal et al., 1995; White et al., 2009). This intrinsic signaling appears in the form of slow varying spontaneous fluctuations in the blood oxygen-level dependent (BOLD) signal in the absence of stimulation. These fluctuations are correlated between brain areas that are anatomically and functionally connected and have been used to map brain functional networks such as sensorimotor, visual, and auditory as well as higher networks such as language and attention (Beckmann et al., 2005; Zhang and Raichle, 2010). These networks are often mapped from data collected at rest and are therefore referred to as Resting State Functional Connectivity (RSFC) maps.

The RSFC analysis can also potentially identify changes in intrinsic neural activity as a result of disease in some neurological and psychiatric conditions. Changes in connectivity strength in different brain networks have been observed in conditions such as autism (Cherkassky et al., 2006), depression (Anand et al., 2005), Alzheimer disease (Li et al., 2002), and attention-deficit hyperactivity disorder (Tian et al., 2006).

Given the advantages of fNIRS, different brain networks have been investigated through RSFC using fNIRS. One of the most common methods for analyzing brain network connectivities using RSFC in fNIRS is cross correlation (Zhang and Raichle, 2010). In cross correlation, an fNIRS channel is selected as the seed channel and the correlations of the signal in all other channels with the seed channel are calculated. The objective is to find the cortical areas whose resting state fluctuations are similar to that of the seed channel. Cross correlation based functional connectivity has been used in conjunction with fNIRS to derive connectivity maps in different brain networks (White et al., 2009; Lu et al., 2010; Duan et al., 2012). Cross correlation and cross coherence of changes in fNIRS channels in particular have been used on infants to study functional connectivity. In a study by Homae et al., the functional connectivity in 3 month old infants with and without audio stimuli was investigated (Homae et al., 2011). The study showed that high correlation exists between neighboring channels and also their corresponding channels in the contralateral hemisphere during resting state. One drawback of correlation based connectivity is that it can be sensitive to detection of spurious connection as a result of presence of cross talk between channels, systemic interference or noise (Tass et al., 1998).

The phase of the oxygenation changes in fNIRS has been shown to contain information on functional connectivity. In a study by Taga et al., strong phase synchronization was observed between cortical regions during sensory processing in young infants (Taga et al., 2011). The phase information was also used in this study to determine the timing and flow of activity in the developing brain. The phase relationship between oxygenated and deoxygenated hemoglobin in resting state in infants was investigated in another study by Taga et al. (2000). Spatially synchronized oscillations were also observed in this study in the measurement channels over the occipital cortex (Taga et al., 2000). Phase based methods have also been used in other modalities such as EEG and MEG for functional analysis (Stam et al., 2007).

Previous studies have investigated the neural connectivity in infants using fNIRS. However, there have been limited studies on the neural systems involved in speech processing in the newborn with some level of inconsistencies in the data regarding the lateralization of the network. In this paper, we have investigated the application of a new method based on the phase relation between spontaneous oxygenated hemoglobin changes in fNIRS channels and have used it as a measure of functional connectivity to identify the speech processing neural systems in newborn infants. Compared to methods based on signal amplitude such as cross correlation, phase is much less sensitive to noise and interferences. It also does not require the assumption of stationarity for the signals. The phase synchronization is not equivalent to coherence or frequency synchronization and is an independent characteristic of the interrelationship between two processes (Tass et al., 1998). To evaluate the feasibility of this analysis method for detecting functional connectivity, we applied it to a study of processing of speech vs. non-speech in newborn human infants. This type of comparison is of particular value for the question being asked as there is a growing body of evidence on the brain areas involved in language processing in neonates, but less on the underlying connectivity.

## 2. Materials and methods

### 2.1. fNIRS data

**Figure 1:**
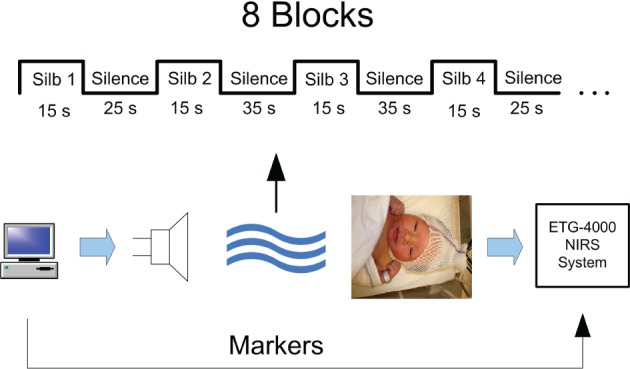
**fNIRS experiment setup**.

The fNIRS data were collected from newborn infants at BC Children's Hospital, Vancouver, Canada, during a separate language perception study (May et al., 2012). Informed consent was obtained from parents when the experiment was being conducted. The study design was approved by the ethics committee of the University of British Columbia. The experiment design and setup are shown in Figure 1. A total of 19 subjects were used in the analysis (mean age 1.4 days, 7 females, age range 0–4 days) out of which two subjects were excluded due to severe artifacts in the signals and poor data quality resulting from optode displacement during data collection. During the experiment, audio stimuli were administered to subjects while the subjects were in state of quiet rest or sleep. The audio stimuli consisted of blocks of sentences in Spanish and Silbo-Gomero. Silbo-Gomero is a whistled language that is a surrogate language of Spanish (Carreiras et al., 2005; Rialland, 2005) It uses whistles rather than speech, and was developed by shepherds in the Canary Islands to communicate across long distances. Spanish and Silbo-Gomero were selected as both are unfamiliar to the infants, while one is a spoken language and the other is not. Each block was 15 s long followed by 25–35 s of silence. A total of 8 blocks for each stimulus were presented in which each block consisted of continuous speech. The total experiment time was 22–25 min.

**Figure 2:**
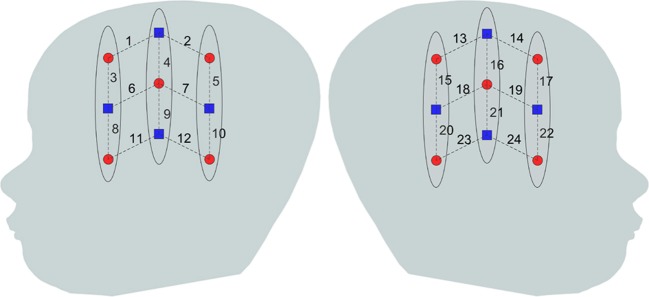
**Optode placement on the head.** Blue squares and red dots indicate detectors and sources, respectively while the dashed lines indicate the measurement channels and the numbers indicate the channel number.

The subjects' brain hemodynamic response was monitored by a 24 channel fNIRS device (Hitachi ETG-4000 machine with 695 and 830 nm lasers at a power of 0.75 mW, interoptode distance of 3 cm and sampling rate of 10 Hz). Two chevron shaped optode holders were used for securing nine 1 mm fibers to the head. There were a total of 4 detector and 5 source fibers on each holder resulting in 12 recording channels per holder. Figure 2 shows the placement of optodes on the subject's head. Surface landmarks (ears or vertex) were used for the placement of the probe holder over the infants' perisylvian area of the scalp. Channels 11 and 12 in LH and 23 and 24 were ideally placed above the infant's ear. A stretchy cap was used to secure the holders on the infants' head.

### 2.2. Data analysis

In order to determine the phase relation between channels, we first extracted the phase of the signal in each channel using the Hilbert transform. The Hilbert transform converts a real valued signal to a complex one, known as analytic signal, whose real part and phase correspond to the original signal and its derived phase, respectively (Oppenheim et al., 1989). The Hilbert transform of signal *x*[*n*] in the frequency domain is defined as (Oppenheim et al., 1989):

(1) $$Y(e^{j\omega })=-jsgn(\omega )\left(X\left(e^{j\omega }\right)\right)$$

where *X* (*e*^*j*ω^) is the Fourier transform of *x*[*n*] and *sgn*(ω) is the sign function having value of 1 for ω > 0 and -1 for ω > 0. The analytic signal can then be written as

(2) $$x_{a}[n]=x[n]+jy[n]$$

where *y*[*n*] is the inverse Fourier transform of *Y*(*e*^*j*ω^).

We used the joint probability distribution of the phases across channels to describe their connectivity. A common model for probability distribution of phase which is the circular analog of the Gaussian distribution is the Von Mises distribution. The Von Mises probability density function (pdf) is defined as (Mardia and Jupp, 2000):

(3) $$f(\theta |\mu ,\kappa )=\frac{1}{2\pi I_{0}(\kappa )}e^{\kappa \cos(\theta -\mu )}$$

where θ is an angle defined in the interval [−π, π) and *I*_0_(κ) is the modified Bessel function of order 0. The parameter κ is the equivalent of the covariance for the Gaussian distribution and μ is the expected value of the angle. The probability density function of the signal phase in channel *m* conditioned on that of channel *n* can therefore be written as:

(4) $$f(\theta_{m}-\theta_{n}|\mu ,\kappa )=\frac{1}{2\pi I_{0}(\kappa )}e^{\kappa_{mn}\cos(\theta_{m}-\theta_{n}-\mu )}$$

κ_*mn*_ describes the intensity of the phase correlation between signals in channels *m* and *n*. In other words, it shows how much the prior information of θ_*n*_ affects distribution of θ_*m*_. The first moment of the distribution given in Equation 3 can be calculated as (Mardia and Jupp, 2000)

(5) $$\begin{array}{l} m_{1}=E[e^{j\theta }]=\int_{-\pi }^{\pi }e^{j\theta }f(\theta |\mu ,\kappa )d\theta \\ \;=\frac{I_{1}(\kappa )}{I_{0}(\kappa )}e^{j\mu } \end{array}$$

Using the first moment, one can estimate parameter κ by numerically solving the optimization problem

(6) $$\underset{\kappa }{\arg\min}{\left(\left|m_{1}\right|-\left|\frac{1}{N}\sum_{i=0}^{N-1}e^{j\phi_{i}}\right|\right)}^{2}=$$

(7) $$\underset{\kappa }{\arg\min}{\left(\frac{I_{1}(\kappa )}{I_{0}(\kappa )}-\left|\frac{1}{N}\sum_{i=0}^{N-1}e^{j\phi_{i}}\right|\right)}^{2}$$

where *N* is the number of samples in the data segment and φ_*i*_ is the measured phase of the signal at time point *i*. Parameter μ can then be estimated using

(8) $$\mu =∠\frac{1}{N}\sum e^{j\phi_{i}}$$

A close relationship exists between parameters of the distribution and the phase locking value (PLV) which is a common measure used in EEG signal processing to detect functional connectivity through synchronization between channels. PLV is related to the distribution through the magnitude of the first circular moment of the phase distribution (Canolty et al., 2012)

(9) $${\text{PLV}}_{mn}=\left|E\left[e^{j(\theta_{m}-\theta_{n})}\right]\right|=\frac{I_{1}(\kappa )}{I_{0}(\kappa )}$$

One advantage of using Von Mises distribution over phase locking for connectivity analysis is that once the parameters are estimated, one would have the distribution function and can re-sample from the distribution to determine the significance levels. Also, the preferred phase difference is not available in PLV.

To evaluate fNIRS functional connectivity, we first calculated the phase from all fNIRS channels using the Hilbert transform as described earlier. Ideally, we would be interested in phase relations between channels when subjects are not exposed to any type of stimulation to reveal intrinsic network activities. It was shown in an earlier study, however, that the infant brain produces no significant response in the language network to Silbo-Gomero stimuli (May et al., 2012). We therefore used the fNIRS data during Silbo-Gomero stimulation as an alternative to resting state. The BOLD spontaneous fluctuations are concentrated at frequencies of less than 0.1 Hz (Biswal et al., 1995). Therefore, the signals were first filtered with an infinite impulse response bandpass filter (IIR) between 0.02 and 0.08 Hz to extract spontaneous hemodynamic activities and reject other interferences. This frequency range is comparable to those used in other studies investigating RSFC using fMRI and fNIRS (Biswal et al., 1995; Duan et al., 2012).

fNIRS data in general can be contaminated with motion artifacts as the result of subjects' spontaneous movements. These artifacts create interference in the form of highly correlated phase changes in fNIRS channels, especially in spatially close channels. This interference results in a very high phase correlation and can obscure underlying phase connections between channels. Even though filtering of the motion artifacts is possible, in order to minimize possibility of introducing any inter-dependence between channels, no artifact removal procedure was applied. Instead, the channels for all subjects were inspected visually and artifact contaminated regions within the Silbo-Gomero stimulation window were marked. An artifact free segment of the data in each channel was then selected for the analysis and the phase of the selected signal segments were then derived using the Hilbert transform. Since the brain shows no response to this stimulation type, the stimulation onsets were ignored and the segments were selected independent of the stimulation onsets. The segments contained variable number of stimulation blocks and their length ranged from 50 to 220 s.

The channel with the highest activation in the grand average for the Spanish stimulation task during the original study in the left hemisphere was selected as the seed channel for the RSFC analysis. The joint phase distribution of the seed channel and all other channels was then estimated by calculating the phase difference between the seed channel and other channels and then estimating κ_*mn*_ and μ_*mn*_ using Equations 7 and 8. We used a simplex derivative-free method to solve Equation 7 and derive κ_*mn*_ (Lagarias et al., 1998). The analysis was performed in MATLAB (Mathworks MA, USA) and the phase coupling estimation toolbox developed by Cadieu et al. was used for parts of the analysis (Cadieu and Koepsell, 2010)^1^.

The analysis was performed on oxygenated hemoglobin (HbO_2_) changes only. Previous studies on the application of fNIRS to detect language network activity and connectivity have shown that the oxygenated hemoglobin is more sensitive to regional cerebral blood flow changes than the deoxygenated hemoglobin with the equipment used here (Hoshi, 2007; Gervain et al., 2008; Lu et al., 2010).

To examine the validity and reliability of the connectivity information derived with this method, we divided the subjects randomly into two groups and evaluated the connections for each group, similar to the approach used in Zhang et al. (2010). We then compared the correlation of the connectivities between the groups.

Other studies have suggested that the language network is left lateralized. We verified this in the network derived using our method. The lateralization was quantified using (Binder et al., 1996; Zhang et al., 2010)

(10) $$LI=\frac{1}{M/2}\sum_{i=1}^{M/2}\frac{\kappa_{L}^{i}-\kappa_{R}^{i^{\prime }}}{\kappa_{L}^{i}+\kappa_{R}^{i^{\prime }}}$$

where M is the total number of channels, κ^*i*^_*L*_ is the value of κ_*is*_ in which *i* is the channel number and *s* is the seed channel in the left hemisphere. κ^*i*′^_*R*_ is the value of the same parameter with the channel symmetric to *i* in the right hemisphere. The significance level of the calculated lateralization index is then evaluated. The lateralization index results in a number between −1 and 1 with more positive numbers indicating higher degrees of left lateralization.

As the final step, we defined 4 regions of interest (ROI), 2 inside the language network and 2 outside the network and evaluated the connection strengths in these areas. In particular, channels 6 and 7 were selected inside the network, based on our prior knowledge that the physical area they cover is in the language network, and channels 1 and 12 outside the network with the optode configuration used in the current study. Channel 1 is over the frontal areas while channel 12 covers the temporal area. The choice of these channels as being outside the language area is justified by the fact that they showed no significant activation in response to native language or Spanish (May et al., 2012).

## 3. Results

**Figure 3:**
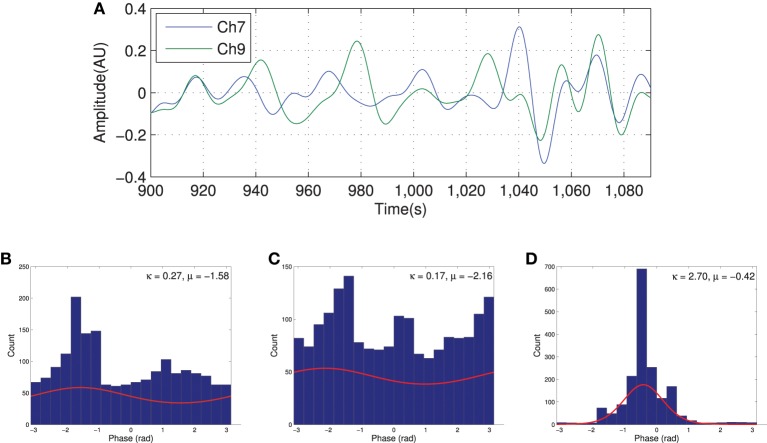
**Filtered HbO_2_ signal recorded from two channels with high degree of connection (A) and the distribution of the phases for each channel [(B) for channel 7 and (C) for channel 9] along with the joint phase distribution (D).** The red curve in histograms shows the estimated probability distribution.

Figure 3 shows the HbO_2_ signal from channels 7 and 9, the seed, for a typical subject. Qualitatively, the histogram of the phase at different time points for both channels does not show a clear dominant phase range as shown in Figures 3B,C. However, the joint distribution histogram shown in Figure 3D has a sharp peak focused around the mean phase difference. This is also evident in the Von Mises distribution function plot derived from estimated parameters in each case (shown in red). The estimated distribution parameters are also indicated in the figure. In the case of the phase histogram for individual channels (Figures 3B,C), the values of estimated κ_*mn*_ are much smaller than in the conditional distribution (Figure 3D). This indicates a high phase relationship between the two channels and is interpreted as connectivity.

**Figure 4:**
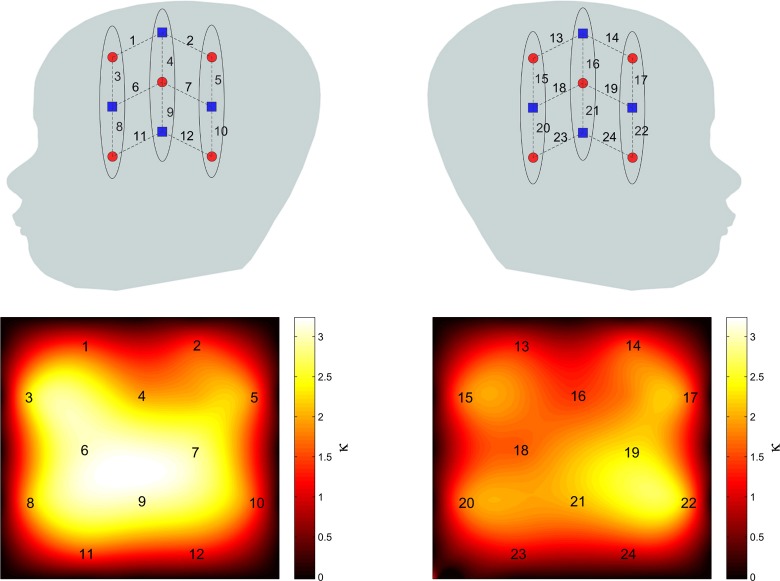
**Group level RSFC maps with channel 9 in the language area used as the seed channel**. **Left** and **right** panels correspond to **left** and **right** hemispheres.

Using the method described earlier, the group level resting state functional connectivity map with channel 9 chosen as the seed channel was derived and is shown in Figure 4. The detected network includes the areas known to be associated with language network including the superior temporal gyrus and Broca's area. The maps are also in agreement with those obtained for the language network in adults using correlation based fNIRS connectivity studies (Zhang et al., 2010).

**Figure 5:**
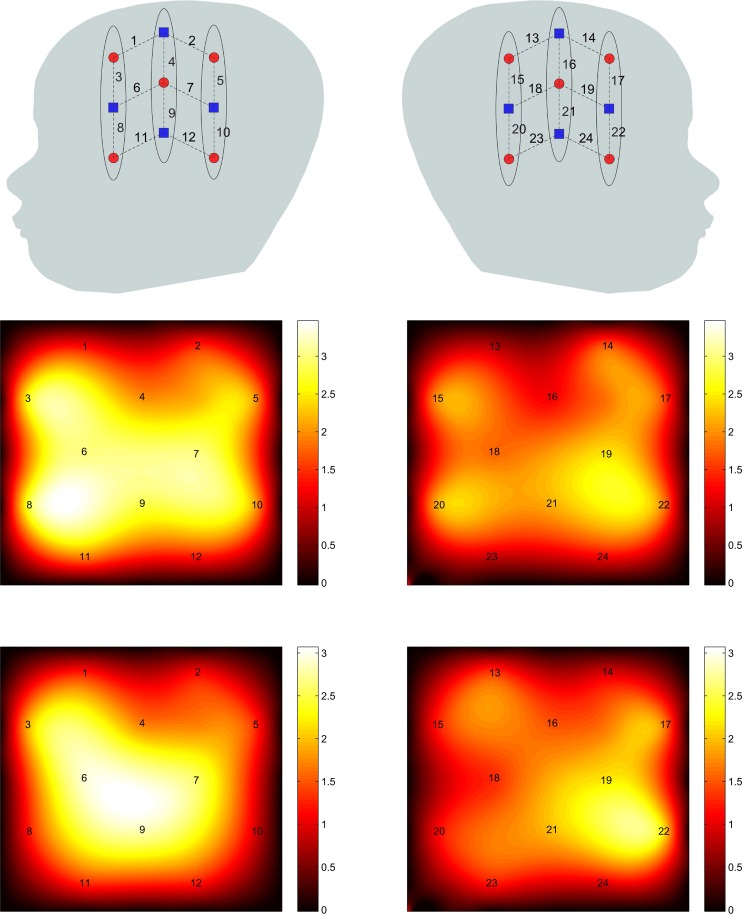
**The connectivity map for the 2 subgroups with channel 9 used as the seed channel.** The **top** and **bottom** panels are results of subgroups 1 and 2, respectively. **Left** and **right** panels correspond to **left** and **right** hemispheres.

**Figure 6:**
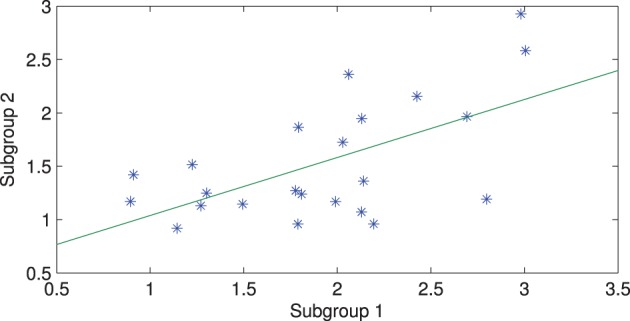
**Correlation between connection strengths in the two subgroups**.

The connectivity maps resulting from the 2 random subgroups are shown in Figure 5. The maps for the two subgroups cover similar areas in both hemispheres. The correlation between individual connections in the two subgroups is shown in Figure 6 (Pearson correlation *r* = 0.6).

**Figure 7:**
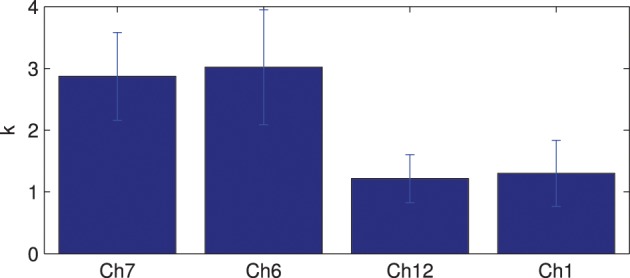
**ROI connections comparison between language area (represented by channels 7 and 6) and outside language area (represented by channels 1 and 12).** The bars indicate the standard error of the mean.

Figure 7 shows the results of ROI connectivity analysis where the connection strength between the seed channel and 2 channels in the language area (6 and 7) is compared with that with two channels outside the language system (channels 1 and 12). Analysis of variance indicates significant difference between the connections (ANOVA *p* < 0.01) inside and outside the language network. In particular, channels 6 and 7 connections are not different while they are both higher than that of channels 12 and 1 in the temporal and frontal areas, respectively. Results of multiple comparison test are shown in Table 1 (Tukey's test).

**Table 1:** **Pairwise comparison between selected ROIs inside and outside language network (Tukey's test)**.

**Channel Pair**	**7-6**	**7-12**	**7-1**	**12-1**
Mean Δκ	−0.15	1.65	1.57	−0.09
CI (95%)	[−1.43 1.13]	[0.37 2.94]	[0.29 2.85]	[−1.37 1.20]

**Figure 8:**
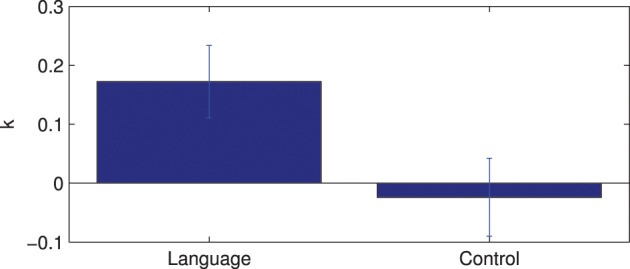
**Lateralization index for the language network and comparison with control network**.

The results of the lateralization analysis are shown in Figure 8. The average lateralization index is 0.172 and is significantly different from zero (1 sample *t*-test, *p* < 0.001). Here, the lateralization index is also compared for all subjects between the language network and a control case. The control network is created by choosing channel 11 as the seed channel. There is a significant difference in lateralization index between the language and control network (paired *t*-test *p* < 0.01). These results suggest strong left lateralization in the detected language network.

## 4. Discussion and conclusion

We evaluated use of phase synchronization to identify resting state functional connectivity in the language system in infants using fNIRS. We used joint probability distribution of phase between fNIRS channels with a seed channel in the language area to estimate phase relations and identify the language system network. Our results indicate the feasibility of this method in identifying the language system. The connectivity maps are consistent with anatomical cortical connections and are also comparable to those obtained from fMRI functional connectivity studies (Perani et al., 2011; Mahmoudzadeh et al., 2013). The results indicate left hemisphere lateralization of the language network.

The brain networks connectivity reveals information about underlying anatomical areas involved in a particular task. In some disease conditions, changes in cortical connections occur (Zhang and Raichle, 2010). Application of connectivity estimating methods to fNIRS enables investigation of such changes in cases where use of fMRI is not possible, such as in infants and extends utilization of fNIRS in wider range of clinical applications.

Use of fNIRS for analysis of functional connectivity offers several advantages over more traditional fMRI based connectivity analysis. Collection of fMRI data from infants and young children under resting condition can be challenging. In contrary, fNIRS is easily applicable to even newborns. Also, in cases where subjects to be tested are immobilized and can not be transferred to an MRI scanner, portable fNIRS systems can replace fMRI for connectivity analysis. One limitation compared to fMRI is the limited penetration depth which means connectivity analysis will be limited to cerebral cortex. Use of phase information and in particular using the method described here has some advantages that can justify its use for functional analysis. In comparison with amplitude based methods such as cross correlation and cross coherence, phase-based methods do not suffer from spurious connections as a result of common source. The method described here can be generalized to estimate the phase distribution function in a multivariate approach (Canolty et al., 2012). In this way, the connectivity parameter between all channels is estimated at once. This eliminates the limitation of bivariate methods in detection of spurious connections.

One limitation of fNIRS is the lack of information of anatomical structure of participants' brains. Due to the lack of an established standard system for processing infant brains, some procedure to localize the channels to the brain may be necessary. The probe set configuration we used in this study primarily allows measurement of activation from anterior temporal to posterior temporal areas, but given infants' small heads, the anterior temporal areas likely extend into frontal regions and the posterior temporal likely extend into parietal regions. Nonetheless, given the variability among infant head sizes and the lack of agreed upon stereotactic maps of infant heads, we chose the most conservative approach of using the general terms we have used, such as anterior and posterior temporal, to refer to the areas measured.

Our results are comparable to similar studies in the literature. In particular Zhang et al. analyzed RSFC in the language system in adults using fNIRS (Zhang et al., 2010). Their results indicated significant RSFC between left inferior frontal and superior temporal cortices which are associated with language system (Zhang et al., 2010). Using fMRI, Fransson et al. studied resting state networks in the infants brain (Fransson et al., 2007). They observed similar networks in the bilateral temporal/inferior parietal cortex which encompasses primary auditory cortex (Fransson et al., 2007). In a study using fMRI on newborn infants, Perani et al. showed strong interhemispheric functional and structural connections between left and right temporal regions and also between the left and right frontal regions (Perani et al., 2011). Homae et al used the cross correlation and spectral coherence to investigate the functional connectivity in infants' brain (Homae et al., 2011). This study reports high correlation between adjacent channels and the corresponding contralateral channels. This is in agreement with our findings of connectivity between neighboring channels in the language area in the left hemisphere and corresponding channels in the right hemisphere using the phase-based method described here.

Other phase-based methods for analysis of functional connectivity have been reported earlier. In a study by Taga et al, the phase information of the oxygenated and deoxygenated hemoglobin was investigated in the presence of audio stimuli (Taga et al., 2011). The relationship between the stimulation and the evoked responses were used to evaluate the temporal features of the response. One important difference of our work is the absence of the stimulation evoked changes for detection of functional connectivity.

The presence of motion artifacts has a significant effect on the connectivity strength results using phase synchrony method presented here. The motion artifact results in in-phase changes across affected channels which may result in stronger phase correlation compared to those resulting from spontaneous neuronal activity. Therefore, care must be taken to ensure segments being processed do not include motion artifacts. Various methods for removal of motion artifacts in fNIRS have been proposed (Cooper et al., 2012; Brigadoi et al., 2014). In this particular study, we used visual inspection to remove motion contaminated data segments instead of using an automated removal technique. An artifact removal method may introduce some level of distortion in the artifact-free regions of the signal and also may not fully remove the artifacts. Therefore, to avoid detection of connectivity as a result of such distortions, an automated approach was not employed. Saturated channels or channels that have lost coupling to tissue due to displacement will also have similar effect.

Our fNIRS data was not collected during strict “resting” state. We used continuous blocks of data in which subjects were listening to a non-speech audio stimulation. No significant activation compared to baseline was observed on this data and was therefore used as the baseline. Some fMRI studies have used a similar approach for mapping RSFC. The study by Greicius et al. on default mode network in Alzheimer's disease patients for example, was performed during a low demand cognitive task (Greicius et al., 2004; Zhang and Raichle, 2010). An alternative approach in the case of task-related data is to regress out the task evoked response from the data before performing RSFC analysis (Fair et al., 2007; He et al., 2007).

The fNIRS signal is known to contain systemic interferences. This includes interference from cardiac pulsation, respiration, cardiovascular autoregulation and heart rate variability. The frequency band for connectivity analysis must be chosen such that it includes the relevant variations caused by the neurovascular coupling while rejecting the frequency bands containing these interferences. The cardiac interference in our study is around 2 Hz and the very low frequency interference (heart rate variability, cardiovascular autoregulation) is around 0.01 Hz. The respiratory fluctuation is around 0.2 Hz. The frequency band we chose for analysis (0.02–0.08 Hz) does not include these interferences and therefore, connectivity detection as a result of these interferences in unlikely.

Bivariate methods in general can result in non-existing spurious connections when there is a propagation of information from one channel to others. A pairwise measure of connectivity will result in detection of connection between all possible connection pairs, ie. direct or indirect. However, since we are only looking to find channels which belong to the same network, use of a bivariate measure of connectivity can be justified. Most methods for functional connectivity mapping in the literature based on fMRI or fNIRS also use seed based methods which is relying on the bivariate concept of finding coherence/correlation between channels with the seed channel (Greicius et al., 2004; Zhang and Raichle, 2010).

In summary, the results of this paper suggest that the proposed method can be used to reveal underlying connectivity patterns of cognitive functions in the resting state through phase relations between hemodynamic changes in different brain regions. The results also indicate a left lateralization in the detected network which suggests the language system may be left lateralized already in newborns. Most fNIRS studies have shown left lateralization of the language network in older infants and in adults. However, data have revealed inconsistencies regarding lateralization of this network in newborn infants, when examining activation in response to language stimulation (Pena et al., 2003; May et al., 2011; Perani et al., 2011; Sato et al., 2011). The results of this study in terms of the lateralization of the network provide further information and evidence for characterization of the language network in newborn infants. Thus, these results significantly add to the theory on the neural foundations of language acquisition by revealing that language processing networks are left lateralized even before language acquisition proper has begun.

### Conflict of interest statement

The authors declare that the research was conducted in the absence of any commercial or financial relationships that could be construed as a potential conflict of interest.

## Footnotes

^1^http://redwood.berkeley.edu/klab/pce.html

## References

[B1] Anand A. Li Y. Wang Y. Wu J. Gao S. Bukhari L. (2005). Activity and connectivity of brain mood regulating circuit in depression: a functional magnetic resonance study. Biol. Psychiatry 57, 1079–1088 10.1016/j.biopsych.2005.02.021 15866546

[B2] Beckmann C. F. DeLuca M. Devlin J. T. Smith S. M. (2005). Investigations into resting-state connectivity using independent component analysis. Philos. Trans. R. Soc. Lond. B Biol. Sci. 360, 1001–1013 10.1098/rstb.2005.1634 16087444 PMC1854918

[B3] Binder J. R. Swanson S. J. Hammeke T. A. Morris G. L. Mueller W. M. Fischer M. (1996). Determination of language dominance using functional MRI: a comparison with the Wada test. Neurology 46, 978–984 8780076 10.1212/wnl.46.4.978

[B4] Biswal B. Yetkin F. Z. Haughton V. M. Hyde J. S. (1995). Functional connectivity in the motor cortex of resting human brain using echo-planar MRI. Magn. Reson. Med. 34, 537–541 10.1002/mrm.1910340409 8524021

[B5] Brigadoi S. Ceccherini L. Cutini S. Scarpa F. Scatturin P. Selb J. (2014). Motion artifacts in functional near-infrared spectroscopy: a comparison of motion correction techniques applied to real cognitive data. Neuroimage 85, 181–191 10.1016/j.neuroimage.2013.04.082 23639260 PMC3762942

[B6] Cadieu C. F. Koepsell K. (2010). Phase coupling estimation from multivariate phase statistics. Neural Comput. 22, 3107–3126 10.1162/NECOa00048

[B7] Canolty R. T. Cadieu C. F. Koepsell K. Ganguly K. Knight R. T. Carmena J. M. (2012). Detecting event-related changes of multivariate phase coupling in dynamic brain networks. J. Neurophysiol. 107, 2020–2031 10.1152/jn.00610.2011 22236706 PMC3331660

[B8] Carreiras M. Lopez J. Rivero F. Corina D. (2005). Linguistic perception: neural processing of a whistled language. Nature 433, 31–32 10.1038/433031a 15635400

[B9] Cherkassky V. L. Kana R. K. Keller T. A. Just M. A. (2006). Functional connectivity in a baseline resting-state network in autism. Neuroreport 17, 1687–1690 10.1097/01.wnr.0000239956.45448.4c 17047454

[B10] Cooper R. J. Selb J. Gagnon L. Phillip D. Schytz H. W. Iversen H. K. (2012). A systematic comparison of motion artifact correction techniques for functional near-infrared spectroscopy. Front. Neurosci. 6:147 10.3389/fnins.2012.00147 23087603 PMC3468891

[B11] Duan L. Zhang Y. J. Zhu C. Z. (2012). Quantitative comparison of resting-state functional connectivity derived from fNIRS and fMRI: a simultaneous recording study. Neuroimage 60, 2008–2018 10.1016/j.neuroimage.2012.02.014 22366082

[B12] Fair D. A. Schlaggar B. L. Cohen A. L. Miezin F. M. Dosenbach N. Wenger U. F. (2007). A method for using blocked and event-related fMRI data to study “resting state” functional connectivity. Neuroimage 35, 396–405 10.1016/j.neuroimage.2006.11.051 17239622 PMC2563954

[B13] Fransson P. Skiöld B. Horsch S. Nordell A. Blennow M. Lagercrantz H. (2007). Resting-state networks in the infant brain. Proc. Natl. Acad. Sci. U.S.A. 104, 15531–15536 10.1073/pnas.0704380104 17878310 PMC2000516

[B14] Gervain J. Macagno F. Cogoi S. Peña M. Mehler J. (2008). The neonate brain detects speech structure. Proc. Natl. Acad. Sci. U.S.A. 105, 14222–14227 10.1073/pnas.0806530105 18768785 PMC2544605

[B15] Greicius M. D. Srivastava G. Reiss A. L. Menon V. (2004). Default-mode network activity distinguishes Alzheimer's disease from healthy aging: evidence from functional MRI. Proc. Natl. Acad. Sci. U.S.A. 101, 4637–4642 10.1073/pnas.0308627101 15070770 PMC384799

[B16] He B. J. Snyder A. Z. Vincent J. L. Epstein A. Shulman G. L. Corbetta M. (2007). Breakdown of functional connectivity in frontoparietal networks underlies behavioral deficits in spatial neglect. Neuron 53, 905–918 10.1016/j.neuron.2007.02.013 17359924

[B17] Homae F. Watanabe H. Nakano T. Taga G. (2011). Large-scale brain networks underlying language acquisition in early infancy. Front. Psychol. 2:93 10.3389/fpsyg.2011.00093 21687461 PMC3110337

[B18] Hoshi Y. (2007). Functional near-infrared spectroscopy: current status and future prospects. J. Biomed. Opt. 12, 062106 10.1117/1.2804911 18163809

[B19] Lagarias J. C. Reeds J. A. Wright M. H. Wright P. E. (1998). Convergence properties of the nelder–mead simplex method in low dimensions. SIAM J. Optim. 9, 112–147 10.1137/S1052623496303470

[B20] Li S. J. Li Z. Wu G. Zhang M. J. Franczak M. Antuono P. G. (2002). Alzheimer Disease: evaluation of a functional MR imaging index as a marker. Radiology 225, 253–259 10.1148/radiol.2251011301 12355013

[B21] Lu C. M. Zhang Y. J. Biswal B. B. Zang Y. F. Peng D. L. Zhu C. Z. (2010). Use of fNIRS to assess resting state functional connectivity. J. Neurosci. Methods 186, 242–249 10.1016/j.jneumeth.2009.11.010 19931310

[B22] Mahmoudzadeh M. Dehaene-Lambertz G. Fournier M. Kongolo G. Goudjil S. Dubois J. (2013). Syllabic discrimination in premature human infants prior to complete formation of cortical layers. Proc. Natl. Acad. Sci. U.S.A. 110, 4846–4851 10.1073/pnas.1212220110 23440196 PMC3607062

[B23] Mardia K. V. Jupp P. E. (2000). Directional Statistics. Chichester, NY: John Wiley

[B24] May L. Byers-Heinlein K. Gervain J. Werker J. F. (2011). Language and the newborn brain: does prenatal language experience shape the neonate neural response to speech? Front. Psychol. 2:222 10.3389/fpsyg.2011.00222 21960980 PMC3177294

[B25] May L. Gervain J. Carreiras M. Werker J. F. (2012). The specificity of the neural response to language at birth, in fNIRS Meeting, London.

[B26] Oppenheim A. V. Schafer R. W. Buck J. R. (1989). Discrete-time Signal Processing. Englewood Cliffs, NJ: Prentice Hall

[B27] Peña M. Maki A. Kovacić D. Dehaene-Lambertz G. Koizumi H. Bouquet F. (2003). Sounds and silence: an optical topography study of language recognition at birth. Proc. Natl. Acad. Sci. U.S.A. 100, 11702–11705 10.1073/pnas.1934290100 14500906 PMC208821

[B28] Perani D. Saccuman M. C. Scifo P. Anwander A. Awander A. Spada D. (2011). Neural language networks at birth. Proc. Natl. Acad. Sci. U.S.A. 108, 16056–16061 10.1073/pnas.1102991108 21896765 PMC3179044

[B29] Rialland A. (2005). Phonological and phonetic aspects of whistled languages. Phonology 22, 237 10.1017/S0952675705000552

[B30] Sato Y. Mori K. Koizumi T. Minagawa-Kawai Y. Tanaka A. Ozawa E. (2011). Functional lateralization of speech processing in adults and children who stutter. Front. Psychol. 2:70 10.3389/fpsyg.2011.00070 21687442 PMC3110423

[B31] Stam C. J. Nolte G. Daffertshofer A. (2007). Phase lag index: assessment of functional connectivity from multi channel EEG and MEG with diminished bias from common sources. Hum. Brain Mapp. 28, 1178–1193 10.1002/hbm.20346 17266107 PMC6871367

[B32] Taga G. Konishi Y. Maki A. Tachibana T. Fujiwara M. Koizumi H. (2000). Spontaneous oscillation of oxy- and deoxy- hemoglobin changes with a phase difference throughout the occipital cortex of newborn infants observed using non-invasive optical topography. Neurosci. Lett. 282, 101–104 10.1016/S0304-3940(00)00874-0 10713406

[B33] Taga G. Watanabe H. Homae F. (2011). Spatiotemporal properties of cortical haemodynamic response to auditory stimuli in sleeping infants revealed by multi-channel near-infrared spectroscopy. Philos. Trans. A Math. Phys. Eng. Sci. 369, 4495–4511 10.1098/rsta.2011.0238 22006903

[B34] Tass P. Rosenblum M. G. Weule J. Kurths J. Pikovsky A. Volkmann J. (1998). Detection of n:m Phase Locking from noisy data: application to magnetoencephalography. Phys. Rev. Lett. 81, 3291–3294 10.1103/PhysRevLett.81.3291

[B35] Tian L. Jiang T. Wang Y. Zang Y. He Y. Liang M. (2006). Altered resting-state functional connectivity patterns of anterior cingulate cortex in adolescents with attention deficit hyperactivity disorder. Neurosci. Lett. 400, 39–43 10.1016/j.neulet.2006.02.022 16510242

[B36] White B. R. Snyder A. Z. Cohen A. L. Petersen S. E. Raichle M. E. Schlaggar B. L. (2009). Resting-state functional connectivity in the human brain revealed with diffuse optical tomography. Neuroimage 47, 148–156 10.1016/j.neuroimage.2009.03.058 19344773 PMC2699418

[B37] Zhang D. Raichle M. E. (2010). Disease and the brain's dark energy. Nat. Rev. Neurol. 6, 15–28 10.1038/nrneurol.2009.198 20057496

[B38] Zhang Y. J. Lu C. M. Biswal B. B. Zang Y. F. Peng D. L. Zhu C. Z. (2010). Detecting resting-state functional connectivity in the language system using functional near-infrared spectroscopy. J. Biomed. Opt. 15, 047003 10.1117/1.3462973 20799834

